# WITHDRAWAL: Cantú Syndrome: A New Case and Evolution of Clinical Conditions During First 2‐Year Follow‐Up

**DOI:** 10.1002/ccr3.70570

**Published:** 2025-06-20

## Abstract

**WITHDRAWAL**: MattiucciA. , GirolomoniG. , CassinaM. , ZollerT. , AntoniazziF. , and SchenaD. , “Cantú Syndrome: A New Case and Evolution of Clinical Conditions During First 2‐Year Follow‐Up,” Clinical Case Reports 11, no. 3 (2023): e6928: 10.1002/ccr3.6928.36873080 PMC9979969

The above article, published online on 02 March 2023 in Wiley Online Library (wileyonlinelibrary.com), has been withdrawn by agreement between the authors; journal Editor‐in‐Chief, Dr. Charles Young; and John Wiley & Sons Ltd. The withdrawal has been agreed to because the consent for publication of identifying details of the patient in this case report has been revoked by the patient's parents.

